# (*E*)-1-(2-Hy­droxy-4,6-dimeth­oxy­phen­yl)-3-(4-meth­oxy­phen­yl)prop-2-en-1-one from *Kaempferia rotunda* Val.

**DOI:** 10.1107/S1600536810042674

**Published:** 2010-10-30

**Authors:** Hasnah Mohd Sirat, Yau Sui Feng, Hazrina Hazni, Khalijah Awang, Seik Weng Ng

**Affiliations:** a Department of Chemistry, Universiti Teknologi Malaysia, 81310 Skudai, Malaysia; b Department of Chemistry, University of Malaya, 50603 Kuala Lumpur, Malaysia

## Abstract

The planar –CH=CHC(=O)– fragment (r.m.s. deviation = 0.074 Å) in the title compound, C_18_H_18_O_5_, connects the planar hy­droxy­dimeth­oxy­phenyl (r.m.s. deviation = 0.039 Å) and meth­oxy­lphenyl (r.m.s. deviation = 0.021 Å) parts. The central fragment forms a dihedral angle of 13.7 (1)° with the hy­droxy­dimeth­oxy­phenyl part and 32.0 (1)° with the meth­oxy­phenyl part. The hy­droxy group forms an intra­molecular hydrogen bond to the carbonyl O atom.

## Related literature

For the isolation of the compound from *Kaempferia rotunda*, see: Sirat *et al.* (2001 ▶); Stevenson *et al.* (2007 ▶).
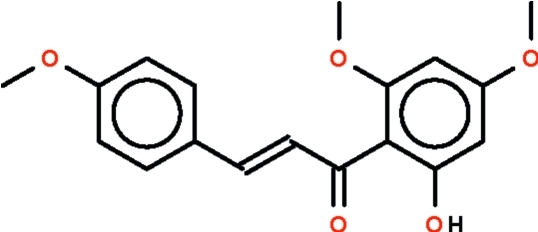


## Experimental

### 

#### Crystal data

C_18_H_18_O_5_
*M* *_r_* = 314.32
Monoclinic, 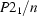

*a* = 12.8502 (8) Å
*b* = 8.3226 (5) Å
*c* = 14.1865 (9) Å
β = 97.765 (1)°
*V* = 1503.29 (16) Å^3^
*Z* = 4
Mo *K*α radiation
μ = 0.10 mm^−1^
*T* = 100 K
0.45 × 0.40 × 0.05 mm

#### Data collection

Bruker SMART APEX diffractometer
13992 measured reflections
3464 independent reflections
2989 reflections with *I* > 2σ(*I*)
*R*_int_ = 0.027

#### Refinement

*R*[*F* ^2^ > 2σ(*F* ^2^)] = 0.036
*wR*(*F* ^2^) = 0.101
*S* = 1.01
3464 reflections
215 parameters
1 restraint
H atoms treated by a mixture of independent and constrained refinement
Δρ_max_ = 0.32 e Å^−3^
Δρ_min_ = −0.23 e Å^−3^

### 

Data collection: *APEX2* (Bruker, 2009 ▶); cell refinement: *SAINT* (Bruker, 2009 ▶); data reduction: *SAINT*; program(s) used to solve structure: *SHELXS97* (Sheldrick, 2008 ▶); program(s) used to refine structure: *SHELXL97* (Sheldrick, 2008 ▶); molecular graphics: *X-SEED* (Barbour, 2001 ▶); software used to prepare material for publication: *publCIF* (Westrip, 2010 ▶).

## Supplementary Material

Crystal structure: contains datablocks global, I. DOI: 10.1107/S1600536810042674/bt5385sup1.cif

Structure factors: contains datablocks I. DOI: 10.1107/S1600536810042674/bt5385Isup2.hkl

Additional supplementary materials:  crystallographic information; 3D view; checkCIF report

## Figures and Tables

**Table 1:** Hydrogen-bond geometry (Å, °)

*D*—H⋯*A*	*D*—H	H⋯*A*	*D*⋯*A*	*D*—H⋯*A*
O1—H1⋯O4	0.87 (1)	1.65 (1)	2.465 (1)	156 (2)

**Fig. 1.**
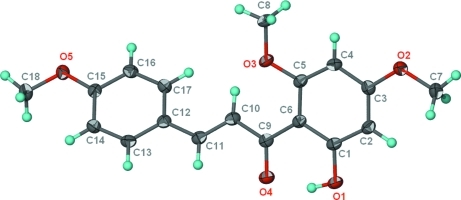
Anisotropic displacement ellipsoid plot (Barbour, 2001) of C18H18O5 at the 70% probability level; hydrogen atoms are drawn as spheres of arbitrary radius.

## supplementary crystallographic information

### Comment

*Kaempferia rotunda* is one of the four Malaysian *Kaempferia* of the Zingiberaceae family; among the constitutents isolated is 1-(2-hydroxy-4,6-dimethoxyphenyl)-3-(4-methoxyphenyl)-2-propen-1-one (Scheme I) (Sirat *et al.*, 2001; Stevenson *et al.*, 2007). The planar –CH═CHC(═O)– fragment (r.m.s. deviation 0.074 Å) connects the planar hydroxyldimethoxyphenyl (r.m.s. deviation 0.039 Å) and methoxylphenyl (r.m.s. deviation 0.021 Å) parts. The fragment is aligned at 13.7 (1) ° with the hydroxydimethoxyphenyl part and at 32.0 (1) ° with the methoxyphenyl part. The hydroxy group forms an intramolecular hydrogen bond to the carbonyl oxygen atom of the fragment (Fig. 1).

### Experimental

*Kaempferia rotunda* rhizomes were purchased from a market in Kempas, Johor. The rhizomes were dried and then grounded. The grounded rhizomes were extracted with *n*-hexane (4.5 *L*), ethyl acetate (4.5 *L*) and methanol (4.5 *L*) in a soxhlet extractor for 16 h. The extracts were concentrated to give a dark brown semi-solid from the *n*-hexane extract (2.32 g), a dark brown oil from the ethyl acetate extract (6.80 g) and a dark brown viscous liquid from the methanol extract (15.27 g). The *n*-hexane extract was purified by column chromatography (93.0 g, column size: 4.5 × 45.0 cm) with *n*-hexane:ether (99:1, 98:2 and 97:3) as eluents to give 20 fractions. Fractions 13 (0.08 g) and 14 (0.02 g) were combined and recrystallized by from an *n*-hexane: ether mixture to afford the title compound (6.9 mg) as yellow crystals.

### Refinement

Carbon-bound H-atoms were placed in calculated positions (C—H 0.95–0.98 Å) and were included in the refinement in the riding model approximation, with *U*(H) set to 1.2–15*U*(C).

The hydroxy H-atom was located in a difference Fourier map, and was refined isotropically with the O–H distance restrained to 0.84±0.01 Å.

### Crystal data

**Table:**

C_18_H_18_O_5_	*F*(000) = 664
*M**_r_* = 314.32	*D*_x_ = 1.389 Mg m^−^^3^
Monoclinic, *P*2_1_/*n*	Mo *K*α radiation, λ = 0.71073 Å
Hall symbol: -P 2yn	Cell parameters from 5893 reflections
*a* = 12.8502 (8) Å	θ = 2.3–28.2°
*b* = 8.3226 (5) Å	µ = 0.10 mm^−^^1^
*c* = 14.1865 (9) Å	*T* = 100 K
β = 97.765 (1)°	Irregular block, yellow
*V* = 1503.29 (16) Å^3^	0.45 × 0.40 × 0.05 mm
*Z* = 4	

### Data collection

**Table:**

Bruker SMART APEX diffractometer	2989 reflections with *I* > 2σ(*I*)
Radiation source: fine-focus sealed tube	*R*_int_ = 0.027
graphite	θ_max_ = 27.5°, θ_min_ = 2.0°
ω scans	*h* = −16→15
13992 measured reflections	*k* = −10→10
3464 independent reflections	*l* = −18→18

### Refinement

**Table:**

Refinement on *F*^2^	Primary atom site location: structure-invariant direct methods
Least-squares matrix: full	Secondary atom site location: difference Fourier map
*R*[*F*^2^ > 2σ(*F*^2^)] = 0.036	Hydrogen site location: inferred from neighbouring sites
*wR*(*F*^2^) = 0.101	H atoms treated by a mixture of independent and constrained refinement
*S* = 1.01	*w* = 1/[σ^2^(*F*_o_^2^) + (0.0545*P*)^2^ + 0.5468*P*] where *P* = (*F*_o_^2^ + 2*F*_c_^2^)/3
3464 reflections	(Δ/σ)_max_ = 0.001
215 parameters	Δρ_max_ = 0.32 e Å^−^^3^
1 restraint	Δρ_min_ = −0.23 e Å^−^^3^

### Fractional atomic coordinates and isotropic or equivalent isotropic displacement parameters (Å^2^)

**Table:**

	*x*	*y*	*z*	*U*_iso_*/*U*_eq_	
O1	0.71889 (6)	0.58291 (10)	0.67517 (6)	0.02051 (19)	
H1	0.7343 (14)	0.536 (2)	0.6242 (9)	0.049 (5)*	
O2	0.44911 (7)	0.93407 (10)	0.74558 (6)	0.0224 (2)	
O3	0.45229 (6)	0.75473 (10)	0.42926 (5)	0.01955 (19)	
O4	0.72544 (6)	0.50207 (10)	0.50890 (6)	0.02089 (19)	
O5	0.61863 (7)	0.54719 (10)	−0.08390 (5)	0.01985 (19)	
C1	0.63266 (9)	0.66965 (13)	0.64416 (8)	0.0166 (2)	
C2	0.58866 (9)	0.75693 (13)	0.71251 (8)	0.0181 (2)	
H2	0.6199	0.7560	0.7771	0.022*	
C3	0.49855 (9)	0.84502 (13)	0.68446 (8)	0.0176 (2)	
C4	0.45027 (9)	0.84618 (14)	0.58972 (8)	0.0177 (2)	
H4	0.3869	0.9041	0.5724	0.021*	
C5	0.49567 (9)	0.76227 (13)	0.52174 (8)	0.0163 (2)	
C6	0.59130 (8)	0.67424 (13)	0.54579 (7)	0.0160 (2)	
C7	0.49545 (11)	0.93606 (16)	0.84345 (8)	0.0257 (3)	
H7A	0.4487	0.9924	0.8815	0.039*	
H7B	0.5632	0.9917	0.8491	0.039*	
H7C	0.5061	0.8255	0.8665	0.039*	
C8	0.35464 (9)	0.83628 (15)	0.40224 (8)	0.0210 (2)	
H8A	0.3332	0.8236	0.3337	0.032*	
H8B	0.3632	0.9507	0.4176	0.032*	
H8C	0.3008	0.7903	0.4370	0.032*	
C9	0.64968 (9)	0.59271 (13)	0.47819 (8)	0.0170 (2)	
C10	0.62915 (9)	0.61886 (14)	0.37466 (8)	0.0180 (2)	
H10	0.5914	0.7110	0.3497	0.022*	
C11	0.66420 (9)	0.51130 (14)	0.31598 (8)	0.0179 (2)	
H11	0.6976	0.4182	0.3446	0.022*	
C12	0.65630 (8)	0.52262 (14)	0.21240 (8)	0.0170 (2)	
C13	0.67036 (9)	0.38511 (14)	0.15961 (8)	0.0194 (2)	
H13	0.6880	0.2870	0.1922	0.023*	
C14	0.65939 (9)	0.38694 (14)	0.06058 (8)	0.0191 (2)	
H14	0.6686	0.2912	0.0262	0.023*	
C15	0.63480 (8)	0.53098 (14)	0.01267 (8)	0.0166 (2)	
C16	0.62576 (9)	0.67210 (14)	0.06464 (8)	0.0192 (2)	
H16	0.6127	0.7714	0.0322	0.023*	
C17	0.63562 (9)	0.66808 (14)	0.16271 (8)	0.0183 (2)	
H17	0.6284	0.7645	0.1971	0.022*	
C18	0.63148 (10)	0.40641 (15)	−0.13935 (8)	0.0219 (2)	
H18A	0.6141	0.4319	−0.2071	0.033*	
H18B	0.5847	0.3215	−0.1222	0.033*	
H18C	0.7045	0.3696	−0.1267	0.033*	

### Atomic displacement parameters (Å^2^)

**Table:**

	*U*^11^	*U*^22^	*U*^33^	*U*^12^	*U*^13^	*U*^23^
O1	0.0192 (4)	0.0242 (4)	0.0175 (4)	0.0058 (3)	0.0000 (3)	0.0029 (3)
O2	0.0276 (5)	0.0221 (4)	0.0174 (4)	0.0066 (3)	0.0027 (3)	−0.0003 (3)
O3	0.0168 (4)	0.0253 (4)	0.0156 (4)	0.0041 (3)	−0.0011 (3)	0.0016 (3)
O4	0.0204 (4)	0.0229 (4)	0.0191 (4)	0.0052 (3)	0.0017 (3)	0.0024 (3)
O5	0.0246 (4)	0.0197 (4)	0.0156 (4)	0.0016 (3)	0.0042 (3)	0.0000 (3)
C1	0.0150 (5)	0.0154 (5)	0.0188 (5)	−0.0015 (4)	0.0007 (4)	0.0041 (4)
C2	0.0200 (6)	0.0183 (5)	0.0152 (5)	−0.0012 (4)	−0.0001 (4)	0.0025 (4)
C3	0.0200 (5)	0.0147 (5)	0.0185 (5)	−0.0016 (4)	0.0044 (4)	0.0006 (4)
C4	0.0166 (5)	0.0172 (5)	0.0191 (5)	0.0017 (4)	0.0018 (4)	0.0037 (4)
C5	0.0168 (5)	0.0159 (5)	0.0156 (5)	−0.0025 (4)	0.0002 (4)	0.0032 (4)
C6	0.0160 (5)	0.0157 (5)	0.0160 (5)	−0.0013 (4)	0.0016 (4)	0.0023 (4)
C7	0.0348 (7)	0.0229 (6)	0.0188 (6)	0.0044 (5)	0.0013 (5)	−0.0027 (5)
C8	0.0170 (5)	0.0242 (6)	0.0207 (5)	0.0033 (4)	−0.0019 (4)	0.0034 (4)
C9	0.0161 (5)	0.0156 (5)	0.0189 (5)	−0.0026 (4)	0.0013 (4)	0.0021 (4)
C10	0.0162 (5)	0.0194 (5)	0.0184 (5)	0.0002 (4)	0.0023 (4)	0.0038 (4)
C11	0.0173 (5)	0.0180 (5)	0.0181 (5)	−0.0008 (4)	0.0011 (4)	0.0031 (4)
C12	0.0142 (5)	0.0198 (6)	0.0171 (5)	0.0005 (4)	0.0022 (4)	0.0012 (4)
C13	0.0199 (6)	0.0167 (5)	0.0211 (6)	0.0025 (4)	0.0011 (4)	0.0030 (4)
C14	0.0187 (5)	0.0179 (6)	0.0206 (5)	0.0021 (4)	0.0024 (4)	−0.0014 (4)
C15	0.0131 (5)	0.0210 (6)	0.0160 (5)	−0.0005 (4)	0.0030 (4)	0.0003 (4)
C16	0.0207 (6)	0.0167 (5)	0.0205 (5)	0.0021 (4)	0.0039 (4)	0.0027 (4)
C17	0.0191 (5)	0.0169 (6)	0.0190 (5)	0.0008 (4)	0.0035 (4)	−0.0005 (4)
C18	0.0253 (6)	0.0220 (6)	0.0193 (5)	−0.0011 (5)	0.0060 (4)	−0.0033 (4)

### Geometric parameters (Å, °)

**Table:**

O1—C1	1.3457 (13)	C8—H8A	0.9800
O1—H1	0.867 (9)	C8—H8B	0.9800
O2—C3	1.3615 (14)	C8—H8C	0.9800
O2—C7	1.4349 (14)	C9—C10	1.4728 (15)
O3—C5	1.3558 (13)	C10—C11	1.3411 (16)
O3—C8	1.4326 (13)	C10—H10	0.9500
O4—C9	1.2618 (14)	C11—C12	1.4623 (15)
O5—C15	1.3644 (13)	C11—H11	0.9500
O5—C18	1.4331 (14)	C12—C13	1.3927 (16)
C1—C2	1.3913 (16)	C12—C17	1.4079 (16)
C1—C6	1.4250 (15)	C13—C14	1.3929 (16)
C2—C3	1.3828 (16)	C13—H13	0.9500
C2—H2	0.9500	C14—C15	1.3932 (16)
C3—C4	1.4023 (15)	C14—H14	0.9500
C4—C5	1.3820 (16)	C15—C16	1.3999 (16)
C4—H4	0.9500	C16—C17	1.3804 (16)
C5—C6	1.4318 (15)	C16—H16	0.9500
C6—C9	1.4619 (15)	C17—H17	0.9500
C7—H7A	0.9800	C18—H18A	0.9800
C7—H7B	0.9800	C18—H18B	0.9800
C7—H7C	0.9800	C18—H18C	0.9800
			
C1—O1—H1	103.7 (12)	O4—C9—C6	119.43 (10)
C3—O2—C7	117.01 (9)	O4—C9—C10	117.29 (10)
C5—O3—C8	117.88 (9)	C6—C9—C10	123.16 (10)
C15—O5—C18	117.20 (9)	C11—C10—C9	119.37 (10)
O1—C1—C2	116.51 (10)	C11—C10—H10	120.3
O1—C1—C6	121.08 (10)	C9—C10—H10	120.3
C2—C1—C6	122.39 (10)	C10—C11—C12	126.67 (10)
C3—C2—C1	118.66 (10)	C10—C11—H11	116.7
C3—C2—H2	120.7	C12—C11—H11	116.7
C1—C2—H2	120.7	C13—C12—C17	117.82 (10)
O2—C3—C2	123.37 (10)	C13—C12—C11	119.40 (10)
O2—C3—C4	115.00 (10)	C17—C12—C11	122.77 (10)
C2—C3—C4	121.63 (10)	C12—C13—C14	122.04 (10)
C5—C4—C3	119.44 (10)	C12—C13—H13	119.0
C5—C4—H4	120.3	C14—C13—H13	119.0
C3—C4—H4	120.3	C15—C14—C13	119.13 (10)
O3—C5—C4	122.78 (10)	C15—C14—H14	120.4
O3—C5—C6	115.74 (10)	C13—C14—H14	120.4
C4—C5—C6	121.46 (10)	O5—C15—C14	124.66 (10)
C1—C6—C5	116.20 (10)	O5—C15—C16	115.70 (10)
C1—C6—C9	118.26 (10)	C14—C15—C16	119.64 (10)
C5—C6—C9	125.53 (10)	C17—C16—C15	120.50 (10)
O2—C7—H7A	109.5	C17—C16—H16	119.7
O2—C7—H7B	109.5	C15—C16—H16	119.7
H7A—C7—H7B	109.5	C16—C17—C12	120.72 (10)
O2—C7—H7C	109.5	C16—C17—H17	119.6
H7A—C7—H7C	109.5	C12—C17—H17	119.6
H7B—C7—H7C	109.5	O5—C18—H18A	109.5
O3—C8—H8A	109.5	O5—C18—H18B	109.5
O3—C8—H8B	109.5	H18A—C18—H18B	109.5
H8A—C8—H8B	109.5	O5—C18—H18C	109.5
O3—C8—H8C	109.5	H18A—C18—H18C	109.5
H8A—C8—H8C	109.5	H18B—C18—H18C	109.5
H8B—C8—H8C	109.5		
			
O1—C1—C2—C3	−178.53 (10)	C5—C6—C9—O4	−170.97 (10)
C6—C1—C2—C3	3.23 (17)	C1—C6—C9—C10	−165.63 (10)
C7—O2—C3—C2	0.85 (16)	C5—C6—C9—C10	13.18 (17)
C7—O2—C3—C4	179.78 (10)	O4—C9—C10—C11	22.06 (16)
C1—C2—C3—O2	179.81 (10)	C6—C9—C10—C11	−162.02 (11)
C1—C2—C3—C4	0.96 (17)	C9—C10—C11—C12	−176.23 (10)
O2—C3—C4—C5	178.59 (10)	C10—C11—C12—C13	−162.90 (11)
C2—C3—C4—C5	−2.46 (17)	C10—C11—C12—C17	17.58 (18)
C8—O3—C5—C4	−0.74 (16)	C17—C12—C13—C14	−3.23 (17)
C8—O3—C5—C6	177.90 (10)	C11—C12—C13—C14	177.23 (10)
C3—C4—C5—O3	178.40 (10)	C12—C13—C14—C15	0.76 (18)
C3—C4—C5—C6	−0.17 (17)	C18—O5—C15—C14	−2.19 (16)
O1—C1—C6—C5	176.25 (10)	C18—O5—C15—C16	177.52 (10)
C2—C1—C6—C5	−5.59 (16)	C13—C14—C15—O5	−177.73 (10)
O1—C1—C6—C9	−4.83 (16)	C13—C14—C15—C16	2.57 (17)
C2—C1—C6—C9	173.33 (10)	O5—C15—C16—C17	176.90 (10)
O3—C5—C6—C1	−174.67 (9)	C14—C15—C16—C17	−3.38 (17)
C4—C5—C6—C1	4.00 (16)	C15—C16—C17—C12	0.84 (17)
O3—C5—C6—C9	6.50 (16)	C13—C12—C17—C16	2.41 (17)
C4—C5—C6—C9	−174.84 (11)	C11—C12—C17—C16	−178.06 (11)
C1—C6—C9—O4	10.22 (16)		

### Hydrogen-bond geometry (Å, °)

**Table:**

*D*—H···*A*	*D*—H	H···*A*	*D*···*A*	*D*—H···*A*
O1—H1···O4	0.87 (1)	1.65 (1)	2.465 (1)	156 (2)
